# Performance in daily activities, cognitive impairment and perception in multiple sclerosis patients and their caregivers

**DOI:** 10.1186/s12883-018-1224-z

**Published:** 2018-12-19

**Authors:** G. Fenu, M. Fronza, L. Lorefice, M. Arru, G. Coghe, J. Frau, M. G. Marrosu, E. Cocco

**Affiliations:** Multiple Sclerosis Center, Binaghi Hospital, ATS Sardegna, Department of Medical Sciences and Public Health, University of Cagliari, via Is Guadazzonis, 2, 09126 Cagliari, Italy

**Keywords:** Patients, Caregiver, Multiple sclerosis, Cognition, Daily activities, Perception, BICAMS, Neuropsychological assessment

## Abstract

**Background:**

The relationship between cognitive assessment results in multiple sclerosis (MS) and performance in daily activities (DAs) remains unclear. Our study aimed to evaluate the relationship between cognitive functions (CF) measured by tests, performance in DAs, and the perception of CF in patients and their caregivers (CG) in MS.

**Methods:**

The Brief International Cognitive Assessment for Multiple Sclerosis (BICAMS) battery was used to evaluate cognitive status. We created an ad hoc questionnaire (DaQ) to assess performance in DAs not requiring specific motor skills. We used the Multiple Sclerosis Neuropsychological Questionnaire (MSNQ) to measure each patient self-judgment and caregiver’s perception of CF.

**Results:**

Forty-nine patients and their caregivers were included in the study. Significant correlations were found between the BICAMS and the DaQ (Symbol Digit Modalities Test (SDMT): *r* = − 0.48, *p* < 0.001; California Verbal Learning Test (CVLT): *r* = − 0.33, *p* = 0.01; Brief Visual Memory Test (BVMT-R): *r* = − 0.42; *p* = 0.002); patients self-judgment (SDMT: *r* = − 0.38, *p* = 0.004; CVLT: *r* = − 0.26, *p* = 0.03); caregiver perception of patient’s CF (SDMT: *r* = − 0.52, *p* < 0.001; CVLT: *r* = − 0.3, *p* = 0.01; BVMT-R: *r* = − 0.42, *p* = 0.002). The difference in perception between the patients and their caregivers was related to patient age (*p* = 0.001) and severity of cognitive impairment (*p* = 0.03).

**Conclusions:**

Cognitive assessment results show a significant correlation with performance in daily activities and with patients and, especially, caregiver perception of cognitive impairment. These data support the importance of a routine evaluation of cognitive function in MS that includes an anamnestic evaluation of patients, and, when possible, consideration of the caregiver’s point of view.

**Electronic supplementary material:**

The online version of this article (10.1186/s12883-018-1224-z) contains supplementary material, which is available to authorized users.

## Background

Multiple sclerosis is a chronic disease involving the central nervous system that is caused by a complex interplay between genetic and environmental factors [1–4]. In addition to motor involvement, other clinical manifestation significantly affects the quality of life of patients and their caregivers [5], including fatigue, pain, dysphagia, psychiatric disorders, and cognitive deficits [5–7].

In recent years, an increasing amount of attention has been paid to cognitive impairment in MS [8]. The availability of diagnostic tools such as the Brief International Cognitive Assessment for Multiple Sclerosis (BICAMS) [9], which can be used in daily clinical practice, has helped to better integrate cognitive function into patient monitoring and the evaluation of disability [10, 11].

MS patients with cognitive impairment experience more difficulty working and in social aspects of life, as well as in adherence to therapy and rehabilitative treatment [8, 12].

The correlation between neuropsychological test results and the actual ability of patient to complete tasks of daily life is a debatable issue [8, 12, 13].

Patients with impairment in cognitive function have greater difficulty carrying out tasks of daily life [13, 14].

Test results, as well as the perception of cognitive deficits by the patient and the caregiver, are affected by multiple factors [15]. Therefore, the role of mood disorders, current pharmacological treatments, and the severity of cognitive deficits have been assessed as possible determinants of subjective perception by the patient and family members regarding cognitive function [15].

Our study aimed to evaluate the relationship between cognitive functions, as measured by neuropsychological tests, performance in daily activities (DAs), and the perception of cognitive impairment in MS patients and their caregivers (CGs).

## Methods

### Subject inclusion and study design

Outpatients with a diagnosis of MS were recruited. For each patient just one caregiver was also included in the study.

Inclusion criteria: MS diagnosis according the 2010 revision of the Diagnostic Criteria [16]., age: 18–65 years; caregiver available to participate in the study. Exclusion criteria were: corticosteroid administration or relapse in the previous 30 days, major comorbidity, intake of drugs with activity on the central nervous system, physical disability that did not permit neuropsychological evaluation (i.e., blindness).The caregivers were classified on the basis of the relationship with the patients to confirm the consistency as a privileged informant.

Principal demographic and clinical features for patients included in the study, including sex, age at inclusion, years of education, age at disease onset, and disability (as evaluated by the EDSS scale [17]) were recorded. For the caregiver, we established the type of connection with the patients by classifying them as either partners or family (with their corresponding degree of kinship).

All included subjects signed informed consent form. Even if a significant percentage of included patients have cognitive impairment, the loss of the ability to express consent has been found in no case. In this case the subject would have been included in the study after acquiring the consent of the legal guardian.The study received approval from the local ethics committee.

### Neuropsychological assessment

Cognitive function of all patients included in the study was evaluated using the Italian version of the BICAMS battery that implemented the normative values for the Italian population and corrections for sex, age, and years of education [18]. The BICAMS includes the Symbol Digit Modalities Test (SDMT) for evaluating the processing speed of information, the California Verbal Learning Test (CVLT-II) for evaluating verbal learning and memory, and the Brief Visual Memory Test-Revised (BVMT-R) for evaluating visual learning and memory. According to the Italian Language validation procedure, the normative data have been established as it follows: raw test scores have been converted to scaled scores using the raw-to-scale-score conversions derived from the normative value. Multiple regression equations derived from the normative values have been applied to compute predicted scores for each patient on the basis of principal demographical features (sex, age, years of education). Predicted scores have been then subtracted from each patient’ s actual scores and the differences divided by the standard deviation of the normative values raw residuals for each measure. Finally, the values have been converted to T scores. The T score is standardised measurement of score. A t score is a type of standard score computed by multiplying a z-score by 10 and adding 50. Thus the T score, the average score is 50, and the standard deviation is 10, and the score shows how many standard deviations the result is from the mean. Result on each neuropsychological measure classified as either intact (*T* > 35) or impaired (*T* ≤ 35). Patients showing at least one altered BICAMS test were classified as cognitive impaired. Patients showing no altered test were classified as cognitive preserved.

The perception of the patient’s cognitive deficits was evaluated using the Multiple Sclerosis Neuropsychological Questionnaire patient version (pMSNQ) and caregiver version (cgMSNQ) [15] for the patient and their caregiver, respectively. In order to evaluate the magnitude of the difference in the perception of CI between patients and caregivers, a specific calculation (cgMSNQ - pMSNQ) was used.

### Performance assessment of daily activities

We created an ad hoc questionnaire (DaQP) to evaluate performance in DAs that do not require specific motor skills (i.e., purchasing flight tickets via the Internet, sending an email, creating a shopping list).

Participants were asked to answer the following question:

“During the last year, how much difficulty did you have carrying out the following activities?”

Participants were asked to select an answer from the following options for each activity:

- No difficulty
- Some difficulties
- Impossible to do it

Scores ranged from 12 (no difficulty carrying out activities) to 36 (impossible to do any of the activities). The full version of the questionnaire is shown in an Additional file 1.

To remove the effect of social context on DAs, we also tested the caregiver’s performance using the DaQ (DaQCG), and then subtracted the patient’s score from that obtained by the caregivers to estimate the impact of MS on DAs (DaQP - DaQCG = cost of MS on DAs).

Depression and anxiety were evaluated using the Beck Depression Inventory [7] and Zung Scale [19, 20], respectively.

We also evaluated the patient’s cognitive reserve using a previously validated tool, the Cognitive reserve index questionnaire (CRIQ) [21] as previous used in studies about cognition in MS [22].

### Statistical analysis

All statistical analyses were performed using SPSS for Mac version 22.0 (SPSS Inc., Chicago, IL, USA).

Firstly, a descriptive analysis was performed summarising patients’ demographic and clinical data as mean for quantitative variables and percentages for qualitative variables.

Pearson Test was used to assess correlation between continuous variable as score of BICAMS test and questionnaire results (MSNQp, MSNQcg, DaQP and DaQCG).

*T*-test was used to compare questionnaire results between the two groups (Cognitively impaired and cognitively preserved patients).

Finally, linear regression analyses were used to evaluate the possible relationship between the different perception of CI (patients/caregiver) and the clinical and demographical features. For all assays, statistical significance was set at *p* < 0.05.

## Results

Forty-nine patients and their caregivers were included in the study. The demographic features of the patients included in the study are as follows: female sex: 37/49 (76.0%); mean age: 43.65 years (SD: 11.9); mean EDSS: 3.24 (SD: 2.06); mean disease duration: 12. years (SD: 7.82); mean years of education: 11.38 years (SD: 4.09); CI (at least one test with T-score < 35) was detected in 27/49 (55.1%) as reported in Table 1. Caregiver included as a follows: 29 partners (59.18%), 19 family caregivers (38.77%).

**Table 1:** Demographic and clinical features of the study patients

	Patients (*n* = 49)
Age (years)	43.65 (S.D. 11.9)
Female sex	38 (76%)
EDSS	3.24 (S.D. 2.06)
Disease duration (years)	12.00 (S.D. 7.82)
Education (years)	11.38 (S.D. 4.09)
Cognitive impaired patients	37 (54%)

Pearson test showed a significant correlation between BICAMS tests T-scores (and the number of altered tests) and both MSNQ versions, patients and caregivers. However the correlation was stronger between BICAMS results and cgMSNQ than pMSNQ as showed in Table 2.

**Table 2:** Pearson Correlation Between the T-score in each BICAMS test, the number of altered tests, and the pMSNQ, cgMSNQ DaQP, DaQP-DaQCG scores

		T-score SDMT	T-score CVLT	T-score BVMT-R	Number of Altered Tests
pMSNQ	Correlation coefficient	−0.381	−0.269	−0.189	0.275
	P value	0.004	0.031	0.097	0.028
cgMSNQ	Correlation	−0.521	−0.338	−0.423	0.562
	P value	< 0.001	0.012	0.002	< 0.001
DaQP	Correlation	−0.487	−0.372	−0.395	0.477
	P value	< 0.001	0.004	0.002	< 0.001
DaQP - DaQCG	Correlation	0.358	0.461	0.256	−0.285
	P Value	0.010	0.001	0.049	0.032

Among the BICAMS tests, the strongest correlations were found between the SDMT T-score and the pMSNQ (*r* = − 3.81, *p* = 0.004) and between the SDMT T-score and the cgMSNQ (*r* = 0.52, *p* = 0.000).

The caregiver’s perception of cognitive deficit showed stronger correlations with the tests than the patient’s perception. The correlation was stronger between the cgMSNQ for all three BICAMS tests (SDMT: *r* = − 0.52, *p* < 0.000; CVLT: *r* = − 0.38, *p* value = 0.012) than the pMSNQ, which was significantly correlated with the SDMT (*r* = − 0.38, *p* = 0.004), and CVLT (*r* = − 0.26, *p* = 0.03), but not with the BVMT-R.

Table 2 shows that the daily activity, assessed by DaQP, showed a significant correlation with the cognitive evaluation assessment, measured by the T score of SDMT, as well as the number of altered tests.

The difference in perception between caregivers and patients (cgMSNQ - pMSNQ) showed a significant correlation with number of altered BICAMS tests (*r* = 0.40, *p* = 0.000), age (*r* = 0.50, *p* = 0.000), and EDSS (*r* = 0.38, *p* = 0.008). Linear regression showed that the difference in perception between patients and caregivers depended mainly on age (*p* = 0.006) and the number of altered tests (*p* = 0.03) (Table 3).

**Table 3:** Linear regression showing the difference between cgMSNQ and pMSNQ as dependent variables, and age, EDSS, and the number of altered tests as dependent variables

Coefficients^a^								
Model		Unstandardized Coefficients		Standardized Coefficients	t	Sig.	95.0% Confidence Interval for B	
		B	Std. Error	Beta			Lower Bound	Upper Bound
1	(Constant)	−23,190	5809		−3.992	.000	−34.920	−11.459
	Age	.418	.144	.430	2.907	.006	.128	.708
	EDSS	.335	.847	.061	.395	.695	−1.377	2.046
	Number of Altered Tests	3.165	1.433	.293	2.208	.033	.270	6.060

*Abbreviations*: *pMSNQ* Patients version of the Multiple Sclerosis Neuropsychological Questionnaire, *cgMSNQ* Caregiver version of the Multiple Sclerosis Neuropsychological Questionnaire

^a^ Dependent Variable: Difference between cgMSNQ and pMSNQ

The burden of cognitive impairment on DAs that do not require motor skills, as evaluated by the difference between DaQCG and DaQP, was significantly correlated with all BICAMS tests and with the number of altered BICAMS tests as reported in Table 2.

T-tests showed significant differences between cognitively impaired (CI) and cognitively preserved patients (not-CI) for the Cognitive Reserve Index Questionnaire, pMSNQ, cgMSNQ, and DaQ results. These results are presented in Table 4.

**Table 4:** Independent samples t-test for evaluating the difference between cognitively impaired patients and cognitively preserved patients in the MSNQ, DaQP, and Cognitive Reserve Index Questionnaire scores

	Cognitively impaired patients	Cognitively preserved patients	P value (t-test)
pMSNQ	25.41(12.43)	16.83 (9.33)	0.008
cgMSNQ	26.64 (13.59)	12.55 (9.71)	< 0.001
DaQP	18.41 (6.99)	14.35 (4.32)	0.016
CRIq	85.43 (9.93)	98.89 (11.76)	< 0.001

*Abbreviations*: *pMSNQ* Patients version of the Multiple Sclerosis Neuropsychological Questionnaire, *cgMSNQ* Caregiver version of the Multiple Sclerosis Neuropsychological Questionnaire, *DaQP* ad hoc questionnaire to evaluating performance in daily activities of MS patients, *CRIq* Cognitive Reserve Index Questionnaire

No correlations were found between anxiety and depression scores and the BICAMS results. However a strong correlation was found between Zung Score and pMSNG (r:0.581, p: 0.001) and also between Beck Score and pMSNQ (r: 0.543, p:0.001). No correlation was found between Zung and Beck scores and cgMSNQ.

## Discussion

Results of our study highlight the complexity of the relationship existing between perceived cognitive deficits and those observed through neuropsychological tools. Consistent with findings of previous studies [15,], we found the caregiver’s perception to correlate more strongly with cognitive deficits than the patient’s self-judgment, which had a less robust but still significant correlation with some objective parameters [23].

One study [24] showed that the reliability of the caregiver version of the MSNQ was greater than the patient version, but we found the patient version of the MSNQ to also be significantly correlated with objective deficits.

These results support the importance to involve the caregiver in the anamnestic evaluation of cognitive deficit. In fact, the caregiver point of view may be a real expression of cognitive deficit more than patients’ perception. This attitude is reflected in previous studies that concluded that self-judgment on cognitive function by patients with multiple sclerosis can be problematic and with a difficult interpretation. Several features as depression, and anxiety could play a role in this self-perception. [25–27]. In fact, also in our study self reported measures of cognitive functions are correlated to depression and anxiety.

In our study, the difference in the perception of cognitive deficiency reported by caregivers and patients correlated with the severity of cognitive deficiency, higher age, and disability. For these reasons, when evaluating patients with such characteristics, the caregiver’s view of cognitive impairment should be evaluated with even greater attention.

Equally important is the use of specially developed instruments in the cognitive function anamnesis.

In our study, the BICAMS results were correlated with DAs, which is similar to the findings of a previous study [13] that reported a strong correlation between the BICAMS test results and performance in daily activities (evaluated using computerized tools) in 41 MS patients.

Among the BICAMS tests, SDMT had the strongest correlation with DAs. These results confirm a role for the SDMT in the principle battery of tests used for MS neuropsychological assessment and as a possible screening test for MS. The correlation between assessment results and the ability in tasks in daily activities support the importance of a routinely cognitive function assessment in daily clinical practice. Moreover we found a significant difference in cognitive reserve score between preserved and impaired patients, as in previous study [22], also our data suggested that cognitive reserve could play a role in the complex interplay between structural damage and cognitive functions in multiple sclerosis.

Our study had several limitations. First, an Italian version of the MSNQ is not available.

The original version was translated by two Italian experts and then by a native English speaker. The translated MSNQ was then administered to 5 patients, 5 caregivers, and 5 healthy volunteers (not included in the study) to evaluate the presence of any difficulties in its ability to be understood.

However, a validation process is needed prior to MSNQ testing in a large Italian population. Despite these issues, we preferred using an instrument such as the MSNQ, i.e., one specifically developed for the perception of cognitive deficiencies in MS by patients and caregivers, for the purpose of our study.

Likewise, for the evaluation of performance in DAs for both the patient and the caregiver, we did not find appropriate tools in the literature to allow us to adequately evaluate activities that require the involvement of cognitive function without the need for motor skills; thus, we built an ad hoc test. Activities could be an area of intervention without complications and additional costs.

Another limitation of the study is the use of a short evaluation battery such as BICAMS, an instrument widely used in everyday clinical practice. However, recent evidence highlighted a correlation with more extensive batteries [28].

## Conclusions

In conclusion, although our findings require further study in order to be generalized, they offer several insights as to the perception of cognitive deficits in MS and the correlation between objective cognitive deficits and the actual impact on activities in daily life.

## Additional file

Additional file 1: Full version of the DaQ questionnaire. (DOCX 15 kb)

## Abbreviations

BICAMS: Brief International Cognitive Assessment for Multiple Sclerosis
BVMT: Brief Visual Memory Test-Revised
CFs: Cognitive Functions
cgMSNQ: Multiple Sclerosis Neuropsychological Questionnaire- caregiver version
CGs: Caregivers
CI: Cognitive impairmet
CVLT: California Verbal Learning Test
DaQ: Daily Activities Questionnaire
DaQCG: Daily Activities Questionnaire- caregiver version
DaQP: Daily Activities Questionnaire-patient version
DAs: Daily Activities
MS: Multiple Sclerosis
pMSNQ: Multiple Sclerosis Neuropsychological Questionnaire-patient version
SDMT: Symbol Digit Modalities Test
