# Supplementary material for: Performance in daily activities, cognitive impairment and perception in multiple sclerosis patients and their caregivers
Source: BMC Neurol. 2018 Dec 19;18:212. doi: 10.1186/s12883-018-1224-z (PMC6299565; doi:10.1186/s12883-018-1224-z)
Supplement: Supplementary file 1 — Full version of the DaQ questionnaire. (DOCX 15 kb) [file 12883_2018_1224_MOESM1_ESM.docx]

**Additional File: Full version of the DaQ questionnaire**

During the last year, how much difficulty did you have carrying out the following activities?

**Select an answer for each activity:**

No difficulty`

Some difficulty

Impossible to do it

**Activities:**

Buy a plane ticket online

Find a place on a map of a new city

Keep accounts for your household expenses

Organize and plan your week's personal commitments

Send an email

Find a cooking recipe for a particular dish

Make a shopping list

Make a payment using a bank transfer form

Make a withdrawal from an ATM

Use social networks such as Facebook or Instagram

Take a picture and send it using a mobile phone

Check the time of a movie at the cinema, a TV show, or the result of a sport match using the Internet

**Scores ranged from 12 (**no difficulty carrying out the activities**) to 36 (**impossible to do any of the activities)
